# Insulin Resistance and Vitamin D Deficiency: A Link Beyond the Appearances

**DOI:** 10.3389/fcvm.2022.859793

**Published:** 2022-03-17

**Authors:** Valentina Trimarco, Maria Virginia Manzi, Costantino Mancusi, Teresa Strisciuglio, Ilaria Fucile, Antonella Fiordelisi, Emanuele Pilato, Raffaele Izzo, Emanuele Barbato, Maria Lembo, Carmine Morisco

**Affiliations:** ^1^Department of Advanced Biomedical Sciences, University of Naples Federico II, Naples, Italy; ^2^Department of Cardiac Surgery, School of Medicine, University of Naples Federico II, Naples, Italy

**Keywords:** type 2 diabetes, metabolic syndrome, arterial hypertension, physical exercise, cardiovascular risk, cardiovascular prevention

## Abstract

Vitamin D is a steroid hormone that plays a key role in the regulation of body homeostasis, including cardiovascular function. Although the chronic deficiency of vitamin D is associated with cardiovascular risk factors, as well as with an adverse prognosis, randomized controlled trials have failed in demonstrating that dietary vitamin D supplementation could ameliorate the prognosis of patients with cardiovascular diseases, and suggested that vitamin D deficiency is the expression of the effects of other determinants of cardiovascular risk. Thus, the supplementation of vitamin D is not sufficient to improve the cardiovascular risk profile and prognosis. Insulin resistance is a complex phenomenon that plays a key role in the pathogenesis of conventional cardiovascular risk factors. Interestingly, defects of vitamin D and insulin resistance have a superimposable epidemiological distribution. According to the common view, Insulin resistance is considered the direct or indirect consequence of vitamin D deficiency. However, it is also reasonable to speculate that the deficit or the impaired action of vitamin D, in some circumstances, could be the result of the same pathogenic mechanisms responsible of insulin resistance development. In this case, vitamin D deficiency could be considered an epiphenomenon of insulin resistance. Insulin resistance is a reversible condition, being possibly ameliorated by physical activity and hypocaloric diets. Notably, both physical exercise and energy-restricted dietary regimens are associated with an increase of vitamin D levels. These findings indicate that improving insulin resistance condition is a necessary step to ameliorate vitamin D supplementation-based strategies in cardiovascular prevention.

## Introduction

One of the most controversial aspects of modern medical literature is represented by the role that vitamin D has in cardiovascular (CV) prevention. In fact, several epidemiological studies have reported that the deficiency of vitamin D is associated with conventional CV risk factors, as well as with a high rate of major CV events, and with adverse CV prognosis (1, 2). On the other hand, observational studies, randomized controlled trials (RCT), and meta-analyses of RCT have failed to demonstrate that dietary vitamin D supplementation is able to ameliorate the prognosis of CV diseases (3–5). Several study limitations can account for these conflicting results. In particular, in the majority of trials the value of vitamin D was detected in basal conditions, whereas was not measured at the end of treatment. Furthermore, in diverse studies, different doses and preparations of vitamin D supplements were used and supplement duration was heterogeneous. Finally, the differences in the designs, in the sample size, in the clinical characteristics of the patients enrolled in trials further contributed to generate inconsistent results. However, it is also reasonable to speculate that vitamin D deficiency, rather than being an independent risk factor, could be the expression of the effects of other determinants of CV risk, compromising the availability and/or the biological activity of the vitamin. In general, it is possible to assert that deficiency of vitamin D is a hallmark of poor healthy condition (6). If this is the case, the supplementation of vitamin D is necessary, but not sufficient to ameliorate CV risk profile and prognosis. Thus, the identification and the correction of concomitant pathogenic mechanisms that impair vitamin D action is required to improve vitamin D-based strategies in CV prevention.

Experimental and clinical studies have clearly documented a close relationship between vitamin D deficiency and insulin resistance (IR). IR is a complex phenomenon that plays a key role in the pathogenesis of conventional CV risk factors such as obesity, metabolic syndrome (MS), arterial hypertension, type 2 diabetes (T2D), non-alcoholic fatty liver disease (NAFLD) (7–10). Moreover, IR is involved in the development of asymptomatic organ damage such as left ventricular hypertrophy (LVH), atherosclerosis, and chronic kidney diseases (CKD) (11–13) and in the determinism of CV outcome (14–16). IR is due to an insulin receptor or post-receptor defect, that compromises the hormonal signal transduction mechanisms (17). Notably, insulin receptor is ubiquitously expressed, and insulin exerts not only metabolic effects, but regulates also different biological functions such as cell cycle, neuro-hormonal homeostasis, vascular reactivity, platelet aggregation, ion exchanges and transport (17–19). In addition, IR phenomenon can be organ and/or tissue specific. Therefore, IR, rather than being viewed as a merely metabolic disorder, should be considered as a cluster of abnormalities that impairs several physiological functions.

Vitamin D is a steroid hormone that exerts its effects through vitamin D receptors (VDRs), belonging to the steroid/thyroid receptor family. Like insulin receptors, VDRs are ubiquitously expressed. The binding of the vitamin to its receptor, promotes the translocation of the complex from cytosol into the cellular nucleus, where it interacts with the retinoid x receptors (RXRs). In the nucleus, the heterodimers VDR/RXR bind to the vitamin D response element (VDRE), that, in turn, modulates transcriptional activities of the target genes (20, 21). More than 200 genes (almost 3% of human genome) are up- or down-regulated by vitamin D (22). Actually, vitamin D modulates not only bone metabolism and mineral homeostasis, but also cell cycle, cell proliferation and cell adhesion, immune and inflammatory responses, neuro-hormonal activity, matrix homeostasis, redox status, etc. In addition, VDRs are also expressed on cells membrane. When the ligand binds to VDR on the cell surface, it promotes the activation of several intracellular second messengers, controlling the activity of different kinases such as PKA, PKB, MAPK, etc. These molecular pathways mediate the *non-genomic* effects of vitamin D (23). Definitely, vitamin D, similarly to insulin, can be considered a pleiotropic hormone.

Observational studies have documented that both IR and the deficit of vitamin D are features of similar metabolic and CV disorders (24, 25). In the past years, IR has been considered a direct or indirect consequence of vitamin D deficiency. However, it is also reasonable to speculate that the deficit or the impaired action of vitamin D, in some circumstances, could be the result of the same pathogenic mechanisms responsible for IR development. If it is the case, vitamin D deficiency could be considered an epiphenomenon of IR. Thus, dietary vitamin supplementation alone could result ineffective in CV prevention, if not associated with interventions aimed at restoring insulin sensitivity, or, at least, at ameliorating IR condition.

The aim of this review is A) to outline the principal actions of vitamin D on CV system; B) to summarize the most significant clinical findings regarding the link between the deficit of vitamin D and CV risk; C) to report the principal pathophysiological mechanisms that can account for vitamin D deficiency as consequence of IR; and D) to consider the implication of this association in order to improve vitamin D-based strategies in CV prevention.

## Vitamin D and CV Homeostasis

**Figure 1:**
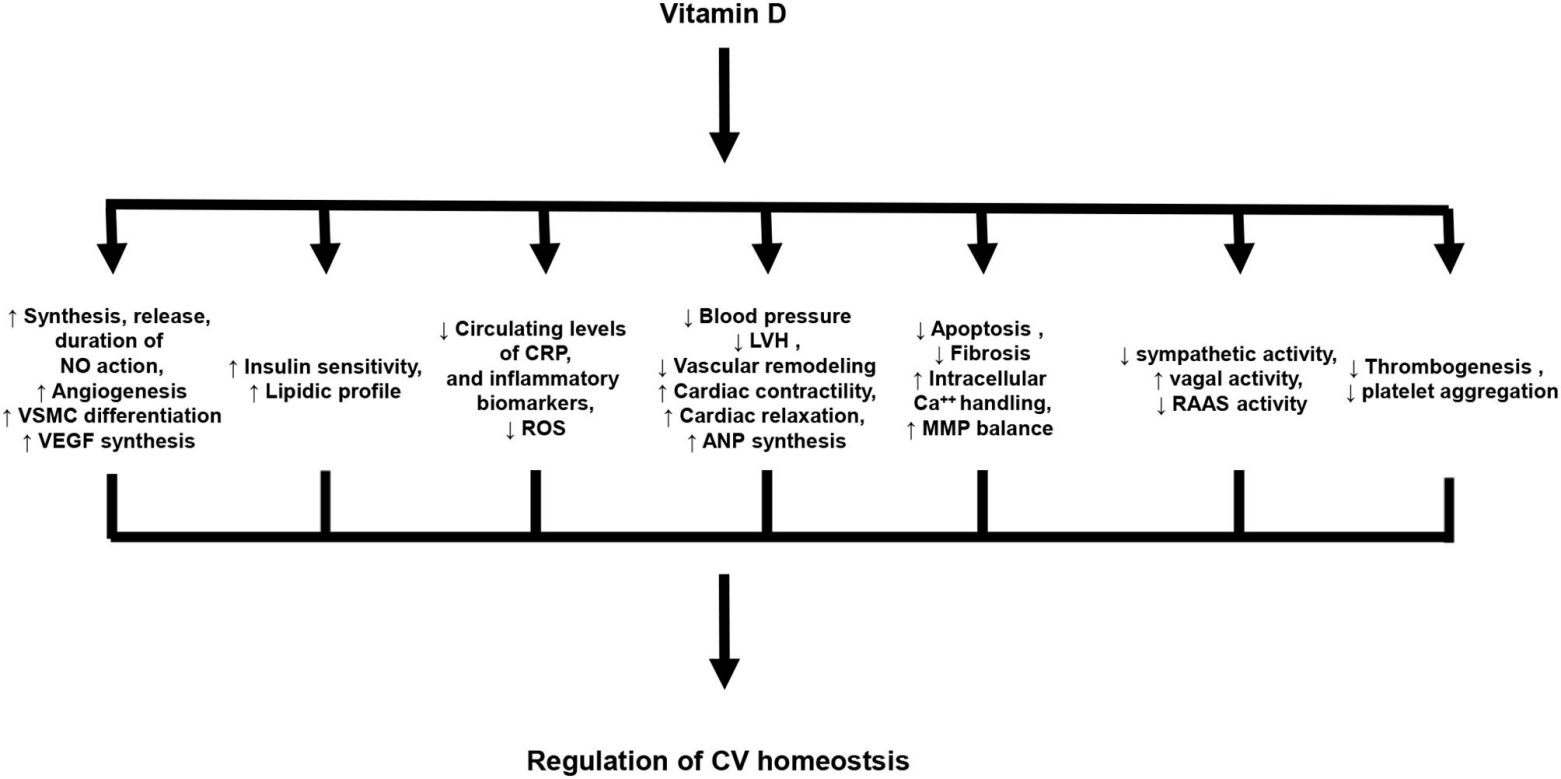
Principal biological effects of vitamin D on cardiovascular system. NO, Nitric oxide; VSMC, vascular smooth muscle cells; VEGF, Vascular endothelial growth factor; CRP, C-reactive protein; ROS, Reactive oxygen species; LVH, Left ventricular hypertrophy; ANP, atrial natriuretic peptide; MMP, Matrix metalloproteinases; RAAS, Renin-angiotensin-aldosterone system.

Experimental data indicate that vitamin D plays a key role in the regulation of CV homeostasis (26). In particular, vitamin D exerts cardio- and vasculo-protective effects, as well as, anti-atherogenic and anti-inflammatory actions. The principal effects of vitamin D in the regulation of CV homeostasis are summarized in Figure 1.

Dysregulation of the renin-angiotensin-aldosterone system (RAAS) plays a pivotal role in the pathogenesis of hypertension, hypertension-induced target organ damage (TOD), CV events, and heat failure (HF) (27, 28). Experimental studies have clearly demonstrated that vitamin D negatively regulates RAAS activity. In particular, in VDR knockout transgenic mice, the different components of RAAS resulted to be upregulated in comparison with wild type control mice. In addition, the cardiac phenotype of these mice was characterized by arterial hypertension and cardiac hypertrophy. These abnormalities were rescued by the administration of vitamin D (29).

The capability of vitamin D to counteract RAAS activity, development of hypertension, and cardiac damage has been confirmed in different experimental studies (30–32). The expression of VDR on cardiac myocytes and fibroblasts suggests that vitamin D plays a relevant role in the regulation of cardiac growth, in physiological as well as in pathological conditions; interestingly, this action results to be independent from hemodynamic forces and neuro-hormonal stimulation. In particular, vitamin D has a protective effect against the development of maladaptive cardiac hypertrophy. Transgenic mice with targeted cardiomyocytes knockout of VDR show, at baseline and after a 7-day infusion of isoproterenol, a greater myocyte size and left ventricular weight/body weight ratio compared with wild type control mice (33). Similarly, the knockout of the gene encoding for 1α-hydroxylase, the enzyme that catalyzes synthesis of the active form of vitamin D, generates a phenotype characterized by enhanced activity of RAAS (21). The cardioprotective effects of vitamin D have been demonstrated also in more complex experimental settings. For instance, in a murine model of left ventricular pressure overload induced by transverse aortic constriction, treatment with *paricalcitol*, a selective agonist of VDR, was documented to prevent the development of left ventricular hypertrophy. This response was associated with the reduction of cardiac fibrosis and the preservation of indexes of left ventricular contraction and relaxation (34). Furthermore, in similar experimental settings, *paricalcitol* was demonstrated to be able to prevent HF worsening and to ameliorate adverse electrophysiological and Ca^++^ handling remodeling, resulting in a reduction of HF-induced arrythmias (35). Noteworthy, vitamin D also exerts a favorable action on both cardiac contractility and relaxation, independently from its anti-hypertrophic action (36–38).

Heart and vasculature represent, at the same time, the sources and target organs of vitamin D. In fact, 1-α-hydroxylase is expressed in cardiac myocytes and in endothelial and smooth muscle cells (SMC) (39, 40). In this context, the autocrine/paracrine activity of vitamin D is extremely relevant. For instance, in mice the selective knock-out of the gene encoding for VDR in the endothelium impairs acetylcholine-induced aortic relaxation, as well as enhances the vasopressor response to angiotensin II, suggesting a mechanistic role of vitamin D in blood pressure (BP) homeostasis and endothelial cell function (41). At a vascular level, the principal effect of vitamin D is an antioxidant action, by superoxide dismutase stimulation (22), counteracting the activity of nicotinamide adenine dinucleotide phosphate (NADPH) oxidase, that promotes the synthesis of reactive oxygen species (ROS). As a consequence of its antioxidant action (42, 43), vitamin D exerts beneficial effects on endothelial function (22, 44), platelet aggregation (45, 46), vascular inflammation (47, 48), thrombogenesis (49, 50), and vascular resistances and remodeling (51–53). Of note, vitamin D plays also a role in the regulation of angiogenesis and vascular repair by the synthesis of vascular endothelial growth factor (VEGF) and cell-derived factor 1 (SDF-1), respectively (54, 55). In addition, vitamin D by the inhibition of macrophages transformation in foam cells antagonizes the development of atherosclerosis (56).

Altogether these experimental data clearly demonstrate the key role of vitamin D in the regulation of CV homeostasis. Therefore, the long-term deficit of vitamin D could be relevant for the pathogenesis of the continuum of CV disease.

## Deficit Of Vitamin D And Cv Risk

Vitamin D deficiency is a worldwide recognized condition. It has been estimated that, in western countries, from one-third to one-half of adult population is affected by mild to moderate vitamin D deficiency (57). It is noteworthy that this defect shares with IR an identical epidemiological distribution. In addition, large cross-sectional and prospective studies have reported an inverse relationship between vitamin D levels and prevalence of CV risk factors and events.

## Deficit Of Vitamin D And Cv Risk Factors

### Diabetes, Metabolic Syndrome, and Obesity

A close relationship was identified between the deficit of vitamin D and T2D. In particular, vitamin D deficiency and severe deficiency are detectable in the 91 and 32% of patients with T2D, respectively (58). In addition, several prospective studies demonstrated that a lower vitamin D status was associated with a higher risk of incidence of T2D. The analysis of two cohorts, the Finnish Mobile Clinic Health Examination Survey and the Mini-Finland Health Survey, including individuals free from diabetes with a follow-up ranging from 17 to 22 years carried out in 1973–1976 and in 1978–1980, respectively, demonstrated that individuals in the highest quartile of serum vitamin D had an 82% lower risk to develop T2D compared with those in the lowest quartile after adjusting for BMI, physical activity, smoking status and education, and thus suggesting that vitamin D may exert a protective effect against incident T2D (59). Similar results were obtained by the analysis of the Nurses' Health Study (60), and the Framingham Offspring Study (61). The role of vitamin D status in the development and progression of T2D has been analyzed by a meta-analysis that evaluated 21 prospective studies, involving 76,220 participants. This analysis demonstrated an inverse and significant association between serum levels of vitamin D and risk of T2D occurrence. In particular, it was documented that each 10 nmol/L increase of vitamin D levels was associated with a 4% lower risk of T2D (62). MS is a cluster of CV risk factors and can be considered a typical feature of IR. Consistently with what affirmed for T2D, low vitamin D status is associated with a higher risk to develop MS. A meta-analysis, aimed at analyzing the risk of developing cardiometabolic disorders by the evaluation of vitamin D serum levels, documented a 51% reduction in risk of MS development for individuals with higher serum concentrations of vitamin (63). Deficiency of vitamin D was also found to be associated with obesity. However, this association was documented by meta-analyses that mainly included cross-sectional and not prospective studies (64).

### Essential Hypertension

Several cross-sectional and longitudinal studies support the notion that vitamin D deficiency is associated with essential hypertension (65). The third national Health and Nutrition Examination Survey (NHANES III), a large cross-sectional study, performed from 1988 to 1994 that analyzed 12,664 individuals representative of the US population, demonstrated an inverse relationship between vitamin D levels and BP values. In particular, SBP was 3 mmHg lower in the group in the highest vitamin D quartile in comparison with the subjects in the lowest quartile (66). A *post-hoc* analysis of the NHANES III showed that high levels of vitamin D (> 32 ng/ml) decreased by 20% the age-induced increase in systolic BP (67). The association between deficiency of vitamin D and incident hypertension was demonstrated in a longitudinal study by the Health Professional Follow-up and the Nurses' Health Study (68) and by the Finnish study (69).

A meta-analysis revising the results of 14 cross-sectional and four longitudinal studies published between 2005 and 2010, including 78,028 individuals, reported an inverse relationship between vitamin D levels and BP. In particular, a decrease of 16 ng/ml in vitamin levels was demonstrated to be associated with an enhanced risk of hypertension by 16% (70). A further meta-analysis performed only on prospective studies, demonstrated that subjects within the top third of baseline vitamin D levels had a 30% lower probability to develop hypertension compared to the bottom third. In particular, the risk to develop hypertension per increment of 10 mg/ml in basal vitamin D levels was 0.88 (71). These results were consistent with the data of a meta-analysis published by Pittas et al. that documented a risk of 80% to develop hypertension in individuals with low serum levels of vitamin D (72). In general, an inverse relationship between vitamin D status and incidence of hypertension has been described.

### Dyslipidemias and Hyperuricemia

There are less and conflicting results about the link between vitamin D status and dyslipidemias. However, vitamin D deficiency was documented to be associated with a worse lipid profile. In a meta-analysis that evaluated 22 cross-sectional studies and 10 RCT, a positive relationship was found between high-density lipoprotein cholesterol (HDL-C), low-density lipoprotein cholesterol (LDL-C) and serum levels of vitamin D. However, the ratio between LDL-C or total cholesterol and HDL-C resulted to be beneficial. In addition, an inverse relationship was found between vitamin D and triglycerides (73). Hyperuricemia has been identified as an independent CV risk factor and often represents a feature of MS. Interestingly, an inverse association between vitamin D status and uric acid levels has been reported. The analysis of the National Health and Nutrition Examination Survey (NHANES) 2007–2014, that included 18.596 individuals, documented that the lowest quartile of vitamin D levels had significative higher risk of hyperuricemia in comparison with the highest quartile (74). These data were consistent with studies on different cohorts and meta-analyses (75–77).

## Deficit Of Vitamin D And Target Organ Damage

Atherosclerosis, LVH, CKD are the principal clinical manifestations of TODs detectable in T2D, hypertension, MS, obesity, etc (78–80). The presence of TOD independently accounts for increased CV risk (81, 82). Experimental and epidemiological data have been depicted a direct association between vitamin D deficiency and occurrence of TODs.

### Atherosclerosis

Atherosclerosis can be considered the paradigm of TODs and is the result of the cross-talk between genetic and environmental factors. Experimental data indicate an association between deficiency of vitamin D and atherosclerosis, and its clinical consequences. Indeed, miniature swine fed with vitamin D–deficient diet for 1 year showed a rapid progression of coronary artery disease by a NFkB-dependent mechanism (83).

Endothelial dysfunction represents the first step of atherosclerotic process. Arterial stiffness is a surrogate marker of endothelial dysfunction (84). There is clear evidence that vitamin D status is inversely associated with impaired arterial stiffness. In fact, in a cross-sectional study that recruited 554 healthy individuals an inverse association between the vitamin D levels and the arterial stiffness was found (85). These data were confirmed in different studies that analyzed different cohorts of subjects. In particular, in a cross-sectional study that recruited 150 postmenopausal women with deficit of vitamin D (<30 ng/ml) an inverse relationship between vitamin D levels and aortic wave velocity was detected, the latter representing an index of aortic stiffness (86). Similarly, in 305 diabetic patients (131 male, and 174 female), enrolled in a cross-sectional study, the association between low levels of vitamin D and increased arterial stiffness was confirmed (87). Consistently, in 52 subjects with uncomplicated end-stage of renal disease (ESRD) a negative correlation was detected between vitamin D status and aortic wave velocity (88). Altogether, these results clearly indicate that vitamin D deficiency is already detectable in the initial phases of the atherosclerotic process.

In addition, epidemiological studies have demonstrated an association between low levels of vitamin D and atherosclerosis in the general population. At this regard, the National Health and Nutrition Examination Survey 2001–2004 evaluated the association between serum levels of vitamin D and prevalence of peripheral artery disease (PAD) in the general US community. PAD was defined by the ankle-brachial index (ABI) <0.9 and the study cohort was categorized according to vitamin D quartiles. The analysis, including 4,839 individuals, documented that low levels of vitamin D were associated with PAD (89). These results were confirmed by the ARIC study. This was a prospective study aimed at identifying the causes of atherosclerosis. The study cohort consisted of 11,789 individuals that were followed-up for 17.1 years. The study population was categorized in three groups according to vitamin D levels: deficient (<20 ng/ml), insufficient (20 to 30 ng/ml) or sufficient (≥30 ng/ml). A Cox regression analysis showed that individuals with deficient values of vitamin D had a higher risk to develop PAD (90). In addition, the association between vitamin D status and atherosclerosis has been reported even in patients with T2D. A cross-sectional study that analyzed 1,018 patients with T2DM documented that PAD gradually increased from patients with the highest to the lowest levels of serum vitamin D. Interestingly, this association remained statistically significant even after adjusting for diabetes-induced risk factors for PAD (91). Similar results were reported for patients with CKD. In fact, in non-dialysis patients with CKD vitamin D deficiency was associated with abnormal ABI. Even in this case the association between PAD and vitamin D status resulted to be independent from CKD-related CV risk factors (92).

Definitely, the strong association between vitamin D deficiency and PAD was documented also by different meta-analyses that revised both prospective and cross-sectional studies (93, 94).

### Left Ventricular Hypertrophy

LVH is an independent risk factor for CV events (95). For many years, the development of LVH has been viewed as an adaptive response of the left ventricle to pressure or volume overload and was considered a typical manifestation of TOD in hypertension and aortic valve stenosis, or renal failure. Nowadays, experimental evidence indicates that LVH development is a complex and multifaceted process that involves not only mechanical forces but also genetic background, neuro-hormonal stimulation, metabolic and anthropomorphic abnormalities, inflammatory response, oxidative stress (96–98). Clinical data are consistent with this notion. In particular, IR-related metabolic and anthropomorphic alterations seem to play a key role in the development of LVH. For instance, it has been documented that low levels of HDL-C are independent determinants of LVH in untreated patients with hypertension (99). Moreover, it has been reported that insulin and insulin-like growth factor 1 (IGF-1) are independent predictors of LVH in patients with hypertension (11). Several reports documented the association of low levels of vitamin D in patients with LVH in different pathological conditions such as hypertension (100–102), aortic stenosis (103) and CKD (104, 105). The contribution of vitamin D deficiency in pathogenesis of LVH is also corroborated by the evidence that the morphology of the left ventricle was preserved in healthy individuals of the Baltimore Longitudinal Study of Aging having normal levels of vitamin D, while the risk of concentric left ventricular remodeling was higher in those with lower vitamin D concentration (106). To note, hyperuricemia, being associated with poor vitamin D status, was found to be a determinant of LVH in subjects with arterial hypertension (107), supporting the notion that vitamin D status plays a leading role in the pathogenesis of CV risk.

### Chronic Kidney Disease

CKD and microalbuminuria are very common features of TODs in hypertension, T2D and MS, and at the same time, are independent determinants of poor outcome in patients with CV diseases (108, 109). Since the kidney is the principal source of the active form of vitamin D, CKD is associated with a severe reduction of the biological activity of the vitamin, without any possibility to regulate its synthesis. In patients with ESRD it has been documented that supplementation of the active form of vitamin D improves survival (110). This result has been confirmed also in patients with CKD not yet treated with hemodialysis (111). Moreover, a strong association between early stages of renal damage and deficiency of vitamin D has been reported. In particular, microalbuminuria and deficit of vitamin D was found in newly diagnosed hypertensive patients (112) and in patients with T2D (113, 114). Interestingly, in patients with T2D, VDR was found to be downregulated and this phenomenon was independently associated with the severity of albuminuria (114). This phenomenon suggests that further pathogenic mechanisms contribute to impair vitamin D action in presence of CKD.

## Deficit Of Vitamin D And Cv Events

There are several large prospective cohort studies that support the notion that the deficit of vitamin D is associated with increased incidence of CV events. The Framingham Offspring Study showed that severe deficiency of vitamin D, during a follow-up period of 7 years (mean 5, 4), was associated with an increase by 62% of incident major CV events, especially in subjects with essential hypertension. This high risk remained unchanged even after adjusting for several confounding factors (115). Similar data were found in the Health Professionals Follow-up Study, reporting a 2 fold increased risk of myocardial infarction in subjects free of CV diseases and with concomitant severe deficit of vitamin D (116), and in the cohort of the Intermountain Healthcare System (1). These data were confirmed in different ethnicities, independently on the distance from the equator and sunlight exposure (117, 118). The association between vitamin D deficiency and the enhanced risk of coronary artery disease and myocardial infarction has been also assessed in a meta-analysis that evaluated 18 prospective studies including a total of 82,982 individuals. This analysis showed that the risk of ischemic heart disease was increased by 39% in the lowest vs. the highest quartile of serum vitamin D levels (119). The deficiency of vitamin D has been also associated with enhanced risk of cerebrovascular events and stroke. The analysis of the Mini-Finland Health Survey Cohort, including 6,219 individuals free from CV diseases at baseline, showed that the lowest quintile of serum levels of vitamin D was predictive of cerebrovascular events (120). This observation was confirmed by several meta-analyses (4, 121).

## Deficit Of Vitamin D And Heart Failure And Mortality

There are incontrovertible data showing that vitamin D deficiency is also associated with poor CV prognosis. In particular, cross-sectional and prospective studies have documented that vitamin deficiency is associated with HF and CV death. The community-based prospective cohort of the Atherosclerosis Risk in Community (ARIC) study showed that the low serum levels of vitamin D (<20 ng/ml) were independently associated with a higher risk to develop HF in 12,215 subjects with a median follow-up of 18 years. In particular, after adjusting for conventional risk factors, the white but not the black individuals within the lowest quintile of vitamin D serum levels had a 27% increased risk of incident HF (122). These data are consistent with those obtained in other study cohorts (123). In this regard, the deficiency of vitamin D (≤ 30 ng/ml) was found in the 89% of patients with HF and concomitant ischemic heart disease (124).

Several studies have also documented that the deficiency of vitamin D is associated with enhanced mortality. The analysis of the Health Maintenance Organization in Israel showed that the deficiency of vitamin D is a significant predictor of reduced survival in patients with HF (125). These results were consistent with data recorded in different study cohorts. In particular, the NHANES III study demonstrated in a cohort of 13,331 individuals followed-up for a median of 8.7 years that the mortality increased by 26% in the lowest quartile of vitamin D (<17.8 ng/ml) in comparison with the highest quartile (89). In addition, vitamin D deficiency was found to be associated with an increased risk of sudden death. A *post-hoc* analysis of the cohort of the Cardiovascular Health Study, including 2,312 participants who were free of clinical CV disease at baseline and were followed-up for a median of 14 years, documented that low levels of vitamin D were associated with a 2-fold increased risk of sudden cardiac death (126). These data were also documented in patients with coronary artery disease and with end-stage of CKD (127, 128).

Definitely, cross-sectional and prospective studies clearly indicate that vitamin D deficiency (below 20 ng/ml) is associated with all clinical manifestations of the continuum of CV disease, from the incidence of CV risk factors to the occurrence of major CV events. The evidence that dietary vitamin D supplementation have failed to improve the CV risk profile and CV prognosis seems to support the concept that additional factors are possibly involved in this process.

## Ir A Determinant Of Deficit Of Vitamin D

In the past decades the deficit of vitamin D has been viewed merely as a nutritional defect, and dietary supplementation of the vitamin was used for the prevention and treatment of rickets in children, and osteoporosis and osteomalacia in adults. Nowadays, it is clear that the deficit of vitamin D is a complex and multifaceted phenomenon with different clinical manifestations.

Epidemiological data indicate that chronic deficit of vitamin D parallels with the clinical manifestations of IR. In fact, in Western Countries, vitamin D deficiency is highly associated with aging, obesity, sedentary lifestyle, T2D, hypertension, liver and renal diseases, that are also clinical features of IR (24, 25). The principal causes of the deficit of vitamin D are reported in Table 1. Of note, in many cases these conditions are associated with IR. These data support the notion that the pathogenic mechanisms responsible for IR development can also, at the same time, account for the deficit or impaired action of vitamin D. Interestingly, the available literature has been focused so far on testing the hypothesis that IR is the consequence of vitamin D deficiency (129–132); on the contrary, whether IR affects vitamin D homeostasis has been poorly investigated.

**Table 1:** Principal causes of vitamin D deficiency.

**Reduced UVB exposure (sunscreen use, winter season, cover-up clothing, air pollution)**
Aging^*^
Low food intake of the vitamin
Gastrointestinal malabsorption
Obesity^*^
Dark skin pigmentation
Smoking
Sedentary behavior^*^
Renal diseases^*^
Liver diseases^*^
Altitude
Distance from the Equator

* *Conditions associated with insulin resistance*.

The majority of vitamin D is obtained from the skin as consequence to ultraviolet B radiations (UVB) exposure, while only 30% is derived from the diet (fatty fish, fish oil, tuna, sardines, egg yolks) (133). The first-rate limiting step in the synthesis of the active form of the vitamin is liver hydroxylation of vitamin D2 (ergocalciferol) or D3 (cholecalciferol) by mitochondrial and microsomal 25-hydroxylase in 25-hydroxy vitamin D (25[OH]D), which, in turn, is converted in the proximal convoluted tubule of the kidney by the 1-alpha-hydroxylase in the active form of the vitamin: 1,25-dihydroxy vitamin D (1,25[OH]_2_D) (134, 135). Minor fonts of 1,25[OH]_2_D are cardiac myocytes, vascular smooth muscle and endothelial cells (39, 40). The compromission of vitamin D action can be due to the impaired activity of cellular hydroxylases or to abnormalities in VDR.

IR can be defined as an impaired biological response to insulin, and it is the results of the combination of genetic abnormalities with environmental factors. The hypercaloric diet plays a key role into IR genesis, accounting for the pathogenesis of several diseases, such as obesity, MS, NAFLD, etc. In Wistar rats it has been reported that 6 months of hypercaloric diet induced IR and severe liver steatosis. This effect was associated with a decrease in serum levels of 25[OH]D by 24%, despite an adequate dose of vitamin D was included into the chow. The supplement of vitamin D only in part restored the levels of 25[OH]D (136). These results allow the speculation that the condition of liver steatosis and IR interfere with the synthesis of vitamin D. There are several clinical evidence that documented reduced levels of vitamin D in subjects with liver steatosis or with NAFLD or with non-alcoholic steatohepatitis (NASH) (137–139). To note, in the past years the deficit of vitamin D was interpreted exclusively as a pathogenic mechanism of NALFD or NASH. However, it is also reasonable to evaluate the deficit of vitamin D as a consequence of different forms of liver diseases. This notion is corroborated by experimental and clinical evidence. In particular, it has been demonstrated that high fat diet-induced obesity decreased the hepatic gene expression of 25-hydroxylases in mice (140, 141). Similar data were found in obese subjects, in whom the overweight determined a decreased expression of cytochrome P450 (CYP) 2R1, the main vitamin D 25-hydroxylase, while the weight loss, induced by gastric bypass, increased the expression of CYP 2R1 (142).

The final step of the synthesis of vitamin D is mediated by renal 1-alpha-hydroxylase, expressed in tubular epithelial cells. Experimental and clinical data indicate that chronic kidney disease decreases the levels of the active form of vitamin D by reducing the activity of 1-alpha-hydroxylase (143, 144). There are also clear experimental data demonstrating that IR is able to reduce renal 1-alpha-hydroxylase activity. In fact, it has been reported in Wistar rats that IR, induced by 18 weeks of high fat diet, reduced the activity of 1-alpha-hydroxylase (143). Similar results were obtained in experimental settings characterized by different model of IR, such as aging and obesity (145, 146). Minor sources of vitamin D are the vessels and the heart. IR is also associated with low grade of vascular inflammation, that is responsible of endothelial dysfunction (147). Vitamin D deficiency was reported to be associated with endothelial function impairment as well as with elevated expression of inflammation mediators such as nuclear factor κB (NFkB) and interleukin-6 (IL-6) (85, 148). Although, the pathogenic mechanisms that account for this association were not deeply investigated yet, it is reasonable to speculate that inflammation-induced reduction of vascular 1-α hydroxylase activity may be the molecular mechanism that, in part, account for the link between vitamin D insufficiency and impairment of vascular function.

IR can also interfere with VDR gene expression. Indeed, in subjects with MS and T2D a downregulation of VDR gene expression was described (114, 149). The role of IR in the regulation of gene expression has been corroborated by the evidence that in obese, as well as in lean subjects, the independent predictors of gene expression of VDR in sub-cutaneous fat resulted to be body mass index (BMI) and homeostasis model of assessment for insulin resistance (HOMA-IR) (150).

**Figure 2:**
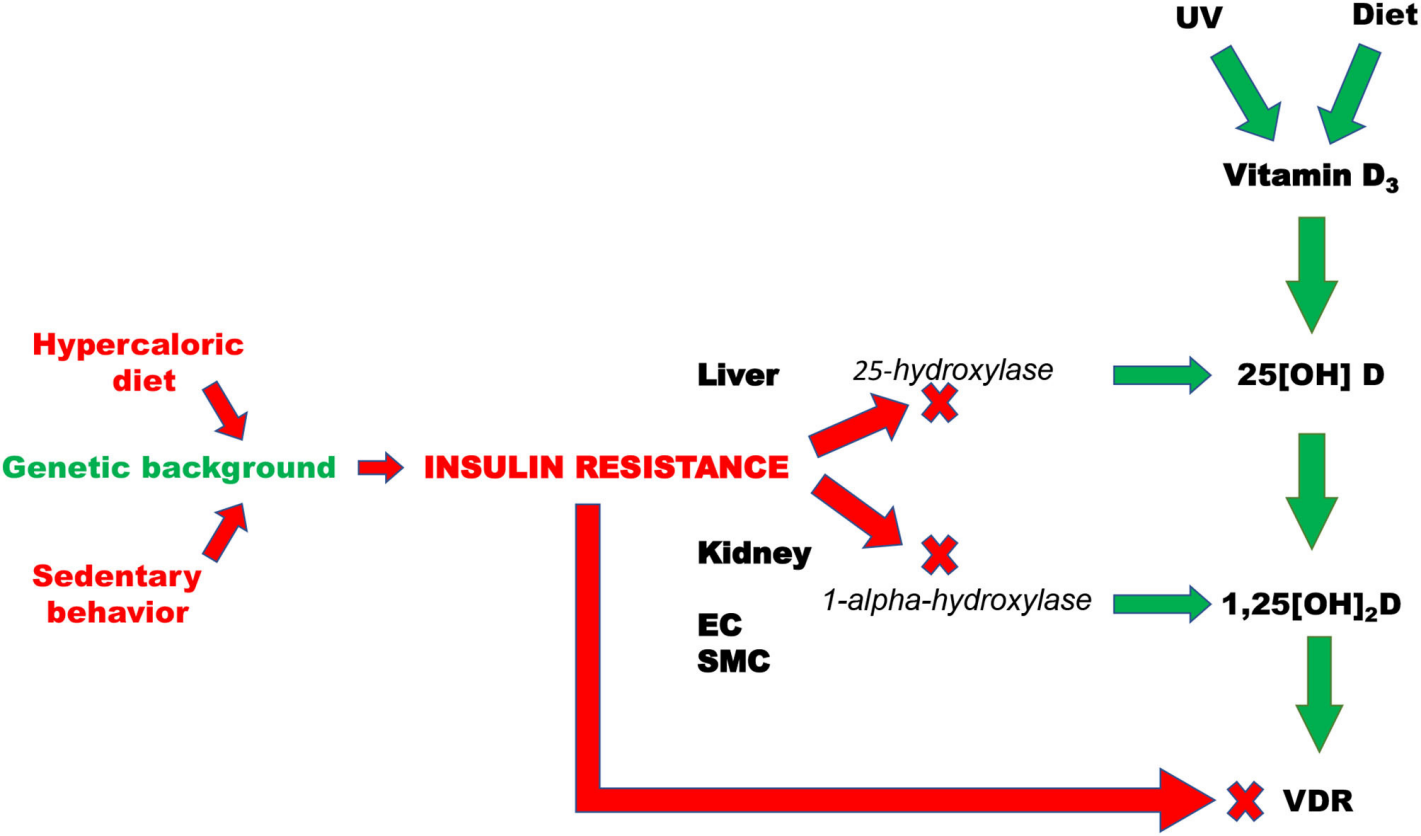
Pathogenic mechanisms that are responsible of insulin resistance-induced deficit of vitamin D. EC, Endothelial cells; SMC, smooth muscle cells; VDR, vitamin D receptor; UV, Ultraviolet radiations.

Altogether these results allow to argue that IR rather than being the consequence, can also play a role as pathogenic determinant of vitamin D deficiency (Figure 2). If this is the case, dietary vitamin D supplementation without any additional intervention aimed at improving insulin sensitivity would be completely ineffective in CV prevention.

## Improvement Of The Vitamin D-Based Strategies In Cv Prevention

In clinical practice vitamin D status is defined by the serum levels of the 25[OH]D, but not by the active metabolite 1,25[OH]_2_D (Table 2). Noteworthy, the current classification of vitamin D status is based exclusively on the consequences detected on bone metabolism. Thus, nowadays, the ranges of vitamin D required to achieve an adequate CV prevention are still undefined (151). Interestingly, subjects with CV risk factors and with CV diseases are not considered individuals at risk for vitamin D deficiency. Thus, the routine assessment of vitamin D status is not indicated in these subjects (152).

**Table 2:** Vitamin D status classification.

**Serum vitamin D concentration (*ng/ml*)**	**Vitamin D status**
≤ 10	Severe deficiency
10–20	Deficiency
20–30	Insufficiency
≥30	Adequate
40–50	Optimal
50–150	Undetermined data
>150	Toxicity

So far, vitamin D supplementation has been viewed as the only treatment option in individuals with vitamin deficiency, and a range of 30–35 ng/ml was proposed to obtain a satisfactory CV prevention (153). However, with the exception of the beneficial effects of supplement of the active form of vitamin D in patients with ESRD, all RCT have failed to demonstrate favorable effects of vitamin supplement on CV outcome (154, 155). On the contrary, there are clear evidence that correction of IR status by both pharmacological and non-pharmacological interventions favorably affects CV risk and prognosis (156–158). Thus, the burning question is whether the improvement of insulin sensitivity contributes to ameliorate the effects of vitamin D supplementation in CV prevention and outcome.

IR and deficiency of vitamin D, with few exceptions, are reversible conditions. In Western countries, physical inactivity and hypercaloric food intake are endemic behaviors. There is clear evidence that exercise training ameliorates insulin sensitivity (159, 160). Notably, physical exercise is associated with an increase of vitamin D levels, independently from sun exposure. In particular, the Third National Health and Nutrition Examination Survey (1988–1994) showed that in old individuals, the frequency of leisure-time physical activity was associated with levels of vitamin D detected in young subjects (161). These data were confirmed by further surveys (2007–2012) (162). Further studies demonstrated the positive effect of physical activity of vitamin D status in different cohorts. The ARIC study demonstrated that in whites but not in black individuals a linear relationship between physical activity and vitamin D levels was described (163).

Although a possible mechanism of exercise-evoked improvement of vitamin D status can be identified in the release of the vitamin from the adipose tissue deposits (164), it is also reasonable to speculate that the improvement of insulin sensitivity can account for this effect. In fact, in an experimental model of T2D, exercise training (swimming) was demonstrated to ameliorate HOMA-IR and metabolic profile, paralleling an improvement of vitamin D status and VDRs expression in skeletal muscle, pancreas and adipose tissue (165).

Similar effects on IR have been also described for dietary habits. The energy-restricted dietary regimens such as the Mediterranean diet, the Dietary Approaches to Stop Hypertension (DASH) diet, the plant-based diets were reported to ameliorate insulin sensitivity and reduce the incidence of T2D (166). In particular, in a *post-hoc* analysis of the Insulin Resistance Atherosclerosis Study cohort, an inverse association between the adherence to the DASH diet and the risk of T2D has been reported (167). Similar results were documented for MS; in this case, the adherence to the DASH diet was associated with a 64% lower risk to develop MS in healthy children and adolescents (168). For the plant-based diets indirect and weaker evidence was reported about the beneficial effects on IR (169). More interestingly, it has been described that the Mediterranean diet can reduce indexes of IR, such as HOMA index (170). In addition, in a cross-sectional study the high adherence to the Mediterranean diet was associated with high serum levels of vitamin D; this association was independent on BMI, waist circumference, physical activity, season and skin pigmentation (171). Similar results were reported in different cohorts of individuals (172, 173), suggesting that the enhancement of insulin sensitivity parallels with the improvement of vitamin D status.

**Figure 3:**
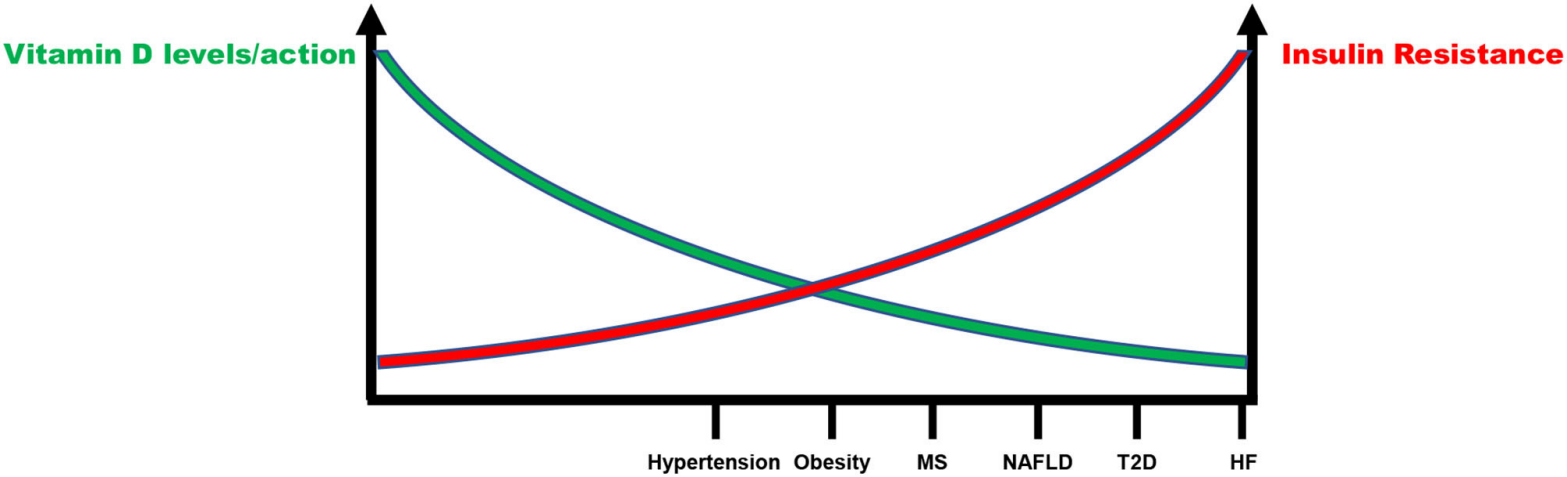
Inverse relationship between insulin resistance and vitamin D status in the continuum of cardiovascular disease. MS, Metabolic syndrome; NAFLD, Non-alcoholic fatty liver disease; T2D, Type 2 diabetes; HF, Heart failure.

**Figure 4:**
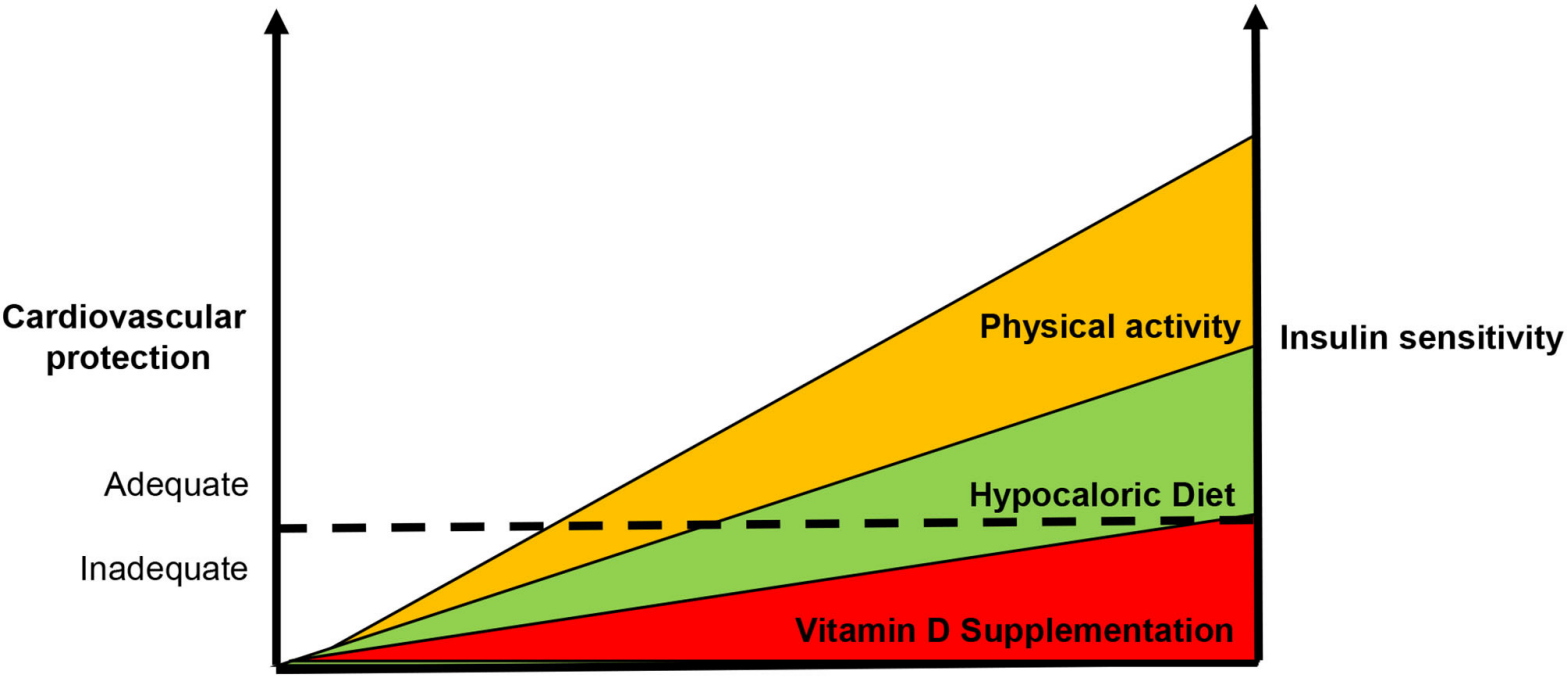
Usefulness of enhanced insulin sensitivity to improve the vitamin d-based strategies in cardiovascular prevention. According our point of view, the supplementation of vitamin D alone is not capable to reduce cardiovascular risk. Since insulin resistance also accounts for the development of vitamin D deficiency, it is necessary to enhance insulin sensitivity in order to improve vitamin D-based strategies in cardiovascular protection. The dot line represents the ideal threshold for the achievement of benefits in terms of cardiovascular prevention.

Definitely, there is an inverse relationship between IR and vitamin D status that account for increased CV risk (Figure 3). Although few data are available, they support the notion that IR correction is necessary in order to optimize the beneficial effects of vitamin D supplementation in CV prevention (Figure 4). CRTs are needed in the next future to clarify this specific issue.

## Author Contributions

VT, ML, and CMo had the idea for this article. RI, MM, CMa, and TS performed the literature search. AF, MM, IF, TS, CMo, and ML drafted the manuscript. EP and EB critically revised the work. All authors contributed to the article and approved the submitted version.

## Conflict of Interest

The authors declare that the research was conducted in the absence of any commercial or financial relationships that could be construed as a potential conflict of interest.

## Publisher's Note

All claims expressed in this article are solely those of the authors and do not necessarily represent those of their affiliated organizations, or those of the publisher, the editors and the reviewers. Any product that may be evaluated in this article, or claim that may be made by its manufacturer, is not guaranteed or endorsed by the publisher.
